# Dietary Approach to Recurrent or Chronic Hyperkalaemia in Patients with Decreased Kidney Function

**DOI:** 10.3390/nu10030261

**Published:** 2018-02-25

**Authors:** Adamasco Cupisti, Csaba P. Kovesdy, Claudia D’Alessandro, Kamyar Kalantar-Zadeh

**Affiliations:** 1 Department of Clinical and Experimental Medicine, University of Pisa, 56126 Pisa, Italy; dalessandroclaudia@gmail.com; 2 Division of Nephrology, Department of Medicine, University of Tennessee Health Science Center, Memphis, TN 38163, USA; ckovesdy@uthsc.edu; 3 Division of Nephrology and Hypertension, University of California Irvine, School of Medicine, Orange, CA 92697, USA; kkz@uci.edu

**Keywords:** chronic kidney disease, dialysis, end-stage renal disease, hyperkalaemia, potassium, diet, nutrition, fibre

## Abstract

Whereas the adequate intake of potassium is relatively high in healthy adults, i.e., 4.7 g per day, a dietary potassium restriction of usually less than 3 g per day is recommended in the management of patients with reduced kidney function, especially those who tend to develop hyperkalaemia including patients who are treated with angiotensin pathway modulators. Most potassium-rich foods are considered heart-healthy nutrients with high fibre, high anti-oxidant vitamins and high alkali content such as fresh fruits and vegetables; hence, the main challenge of dietary potassium management is to maintain high fibre intake and a low net fixed-acid load, because constipation and metabolic acidosis are *per se* major risk factors for hyperkalaemia. To achieve a careful reduction of dietary potassium load without a decrease in alkali or fibre intake, we recommend the implementation of certain pragmatic dietary interventions as follows: Improving knowledge and education about the type of foods with excess potassium (per serving or per unit of weight); identifying foods that are needed for healthy nutrition in renal patients; classification of foods based on their potassium content normalized per unit of dietary fibre; education about the use of cooking procedures (such as boiling) in order to achieve effective potassium reduction before eating; and attention to hidden sources of potassium, in particular additives in preserved foods and low-sodium salt substitutes. The present paper aims to review dietary potassium handling and gives information about practical approaches to limit potassium load in chronic kidney disease patients at risk of hyperkalaemia.

## 1. Introduction

Hyperkalaemia is a common electrolyte abnormality which occurs most frequently in patients with decreased kidney function, with the highest prevalence observed in patients with end-stage renal disease (ESRD). Severe hyperkalaemia is a medical emergency, as high serum potassium levels or its abrupt excursions may be a cause of sudden cardiac death [1,2]. Besides a decrease in potassium excretion by the kidneys (as seen in chronic kidney disease (CKD) or ESRD and often made worse by medications such as inhibitors of the renin-angiotensin-aldosterone system (RAAS)), hyperkalaemia may also be exacerbated by an abnormal redistribution between the intracellular and extracellular space and by increased dietary potassium intake [3,4].

In the early stages of CKD, even very high potassium intake is not sufficient to cause hyperkalaemia and external potassium balance is generally neutral, unless therapies reducing net intracellular shift or renal excretion capacity are administered. This is an important consideration since high potassium diets are useful in patients with CKD because they have been associated with favourable cardiovascular and renal outcomes [5,6]. However, in advanced stages of CKD and in ESRD a positive external potassium balance, namely a dietary input that surpasses output, has a crucial role in engendering hyperkalaemia [3,4], and its prevention requires (among others) a balanced management of dietary potassium load [7,8].

The present paper aims to review dietary potassium handling and gives information about practical approach to limit potassium load in CKD patients at risk of hyperkalaemia.

## 2. Dietary Potassium Intake

Whereas the US Food and Nutrition Board of the Institute of Medicine has set an adequate intake for potassium relatively high in healthy adults, i.e. 4.7 g (120 mmol) per day, the World Health Organization (WHO) recommends a dietary potassium intake of 3.9 g (100 mmol) per day or at least 90 mmol/day (3510 mg/day), to reduce blood pressure and the risk of cardiovascular damage, stroke and coronary heart disease [9]. In patients with non-dialysis dependent (NDD) CKD stages 1–5, the National Kidney Foundation (NKF) suggests an unrestricted potassium intake unless the serum potassium level is elevated. In hemodialysis patients, potassium intake should be up to 2.7–3.1 g/day and in peritoneal dialysis patients close to 3–4 g/day; in both cases, adjustments based on serum potassium levels are crucial [10]. A recent comprehensive review paper on nutritional management of CKD by Kalantar-Zadeh and Fouque [11] has suggested an intake of 4.7 g/day in the early stages of CKD without risk of hyperkalaemia, but a dietary potassium restriction of less than 3 g (less than 77 mmol) per day in CKD patients who tend to develop hyperkalaemia (serum potassium levels >5.3 mEq/L). A low potassium diet is defined as a dietary intake of 2–3 g/day (approximately 51–77 mmol/day) as shown in Table 1.

### 2.1. Renal Regulation of Potassium Balance

The majority of the regulation of potassium balance occurs at the renal level. Following a dietary potassium load, renal excretion increases after a few minutes reaching maximum levels after 2 h [12], thus preventing hyperkalaemia. This occurs by means of increased aldosterone production. Potassium secretion may also be facilitated by a recently postulated enteric sensor that reduces sodium reabsorption in the proximal tubule and facilitates potassium secretion by increased delivery of sodium to the distal tubule [3]. Additional renal responses to potassium loading include reduced sodium reabsorption and increased potassium-channel conductance [3,4]. In patients with advanced CKD the kidneys’ ability to adapt to increased potassium intake diminishes and in ESRD the renal mechanisms of potassium excretion often become negligible, making these patients extremely prone to hyperkalaemia.

### 2.2. The Role of the Gastrointestinal Tract in Regulating Potassium Balance

Dietary potassium is absorbed mostly in the duodenum and jejunum and the net intestinal potassium absorption is approximately 90%. Under physiologic circumstance faecal excretion is quite constant at about 10 mmol/day, with a maximum level of 15–20 mmol/day. The capacity of the colon/rectum to secrete potassium is inversely related to residual kidney function and becomes the main route of potassium excretion in patients with ESRD [13,14].

Potassium concentration in the faeces is very high (83–95 mmol/L), so that diarrhoea (more than 300 cc of fecal volume a day) may lead to profound hypokalemia. Hence, it is conceivable that slow faecal transit time along the intestinal tract favours potassium absorption, whereas faster intestinal transit time reduces potassium absorption [15]. This suggests that constipation, instead of potassium dietary load, is the main determinant of hyperkalaemia in CKD and ESRD patients [16].

### 2.3. Sources, Composition of Diet and Hyperkalaemia

Potassium is present in a large variety of foods, both from animal and plant sources. Potassium is found mainly intracellularly in animals, where it has a crucial role in determining the electric potential of cell membranes and then the excitability of nervous system and muscle cells. Hence it is not surprising that food rich in cells, such as meat or fish, are relevant sources of potassium. Indeed a recent study showed that high protein intake in maintenance dialysis patients has direct correlation with hyperkalaemia [17]. In this study higher dietary potassium intake was associated with increased death risk in long-term haemodialysis patients, even after adjustments for serum potassium level; dietary protein; energy and phosphorus intake; and nutritional and inflammatory marker levels. The potential role of dietary potassium in the high mortality rate of dialysis patients warrants clinical trials.

Notwithstanding the importance of protein intake, fruits and vegetables are supplying the majority of dietary potassium in most diets [18]. Potassium is crucial for many processes in a plant’s life cycle, with its importance considered second only to nitrogen for plant growth and composition (1 to 3% by weight) [19]. Plants require potassium ions for protein synthesis, enzyme activation and maintenance of cation/anion balance in the cytosol. Potassium is also involved in the opening and closing of stomata regulating proton pumps; it plays an important role in photosynthesis and in photo-protection and it takes part in protein synthesis and in downward solute transport from the leaves [19,20]. Potassium is important for crop yield as well as for the quality of edible parts of crops; its deficiency has a strong impact on plant metabolism. Plant responses to low potassium involve changes in the concentrations of many metabolites as well as alteration in the transcriptional levels of many genes and in the activity of many enzymes [19]. Potassium levels in plants are also associated with disease resistance through its effects on decreased cell permeability and decreased susceptibility to tissue penetration. When adequate levels of potassium are present a greater amount of silica is incorporated into the cell walls, strengthening the epidermal layer which represent a physical barrier to pathogens. Moreover, potassium seems to directly contribute to an adequate thickening of cell walls [21]. Purely plant-based LPD (Low Protein Diet) may or may not lead to a more consistent potassium load than animal-based LPD. 

Therefore, in the case of a need for a protein restricted diet in advanced CKD, animal-based LPD could be favoured over vegetarian LPD [22,23], combined with educational strategies to reduce the effective potassium load and close monitoring of serum potassium level. However, such a strategy entails a therapeutic compromise, as it would abandon the additional cardiovascular benefits of a plant-based diet. Plant-based foods have favourable effects on systemic hypertension, on glomerular hemodynamics and perm-selectivity, leading to reduction of proteinuria. They supplylessbio-available phosphorus (in the form of phytate) with favourable effect on the CKD mineral and bone disease (MBD) and they are associated with effective renal protection probably by means of the reduced acid load [24].

However, plant-based foods also supply a high content of fibres and alkali as well as anti-oxidant vitamins and trace elements. Of consequence, a lower net acid load and favourable effects on intestinal motility and microbiota are expected. Prevention or correction of metabolic acidosis and of constipation represent mechanisms that counteract the hyperkalaemia-inducing effects of high potassium intake, and may explain why vegetarian diets, more or less associated with a reduction in protein intake did not induce increase of serum potassium or overt hyperkalaemia in CKD patients [25,26,27,28]. Similarly, during high-fruit intake, no changes in serum potassium levels were reported [23,24,25,27].

Fibre intake has a major role in the modulation of intestinal microbiota, with high-fibre diets promoting the growth of bacteria with saccharolytic metabolism and lowering proteolytic-derived uremic toxins [29,30,31,32] and also leading to faster bowel transit time. Conversely, reduced bowel motility and constipation can induce dysbiosis of the intestinal microbiota, contributing to uremic intoxication and increase net absorption of potassium, leading to hyperkalaemia [16]. Therefore, a high fibre content in the diet should be preserved even when the potassium intake is to be lowered.

## 3. Dietary Intervention to Limit Potassium Intake

In clinical practice, a common dilemma in the management of advanced CKD patients with chronic hyperkalaemia is the patients’ deprivation of the beneficial effects of RAAS inhibitors or of the favourable effects of vegetarian diets [33] in order to control hyperkalaemia. A solution may be the use of intestinal potassium binders [34,35]. However, the capacity of intestinal potassium -binders to remove potassium is limited and they could be expensive in the long run. Therefore, a careful control of the dietary potassium load is in an important aspect of the management of CKD and heart failure patients with, or at risk of hyperkalaemia.

In patients with stage 4–5 NDD CKD and ESRD, dietary potassium management also has to be synchronized with additional nutritional goals, namely the amount of protein intake (restricted in NDD-CKD or increased in ESRD), high fibre intake, reduced net fixed acid production and cardiovascular effects and the favouring of a heart-healthy diet (typically consisting of fruits and vegetables) [36]. 

All of these goals are of clinical relevance while also useful to counteract the risk of hyperkalaemia. To obtain a reduction of potassium load without inducing a decrease in alkali or fibre intake, we recommend the implementation of certain pragmatic interventions:

(a) Knowledge and education about the type of foods which contain excess potassium (per serving or per unit of weight), about the foods needed for proper nutrition in CKD and ESRD, and that supply a low potassium load.
(b) Classification of foods based on their potassium content normalized per unit of fibre.
(c) Education about the use of cooking procedures (especially boiling) in order to achieve demineralization and in particular for removing potassium before eating.
(d) Attention to hidden sources of potassium (e.g., food additives and low-sodium salt substitutes).

The first step is the provision of information about the type of foods which contain excess potassium, which should be avoided. However, potassium is almost ubiquitous, which makes it very difficult for patients to make dietary choices based on general guidance alone. Skilled dietitians are required to properly select foods (Table 2 and Table 3) to individualize dietary programs with low potassium content and to realize intensive educational programs and regular counselling. This activity is time and money consuming and it is difficult to realize in the common clinical practice without the support of dedicated and qualified health professionals.

**Figure 1:**
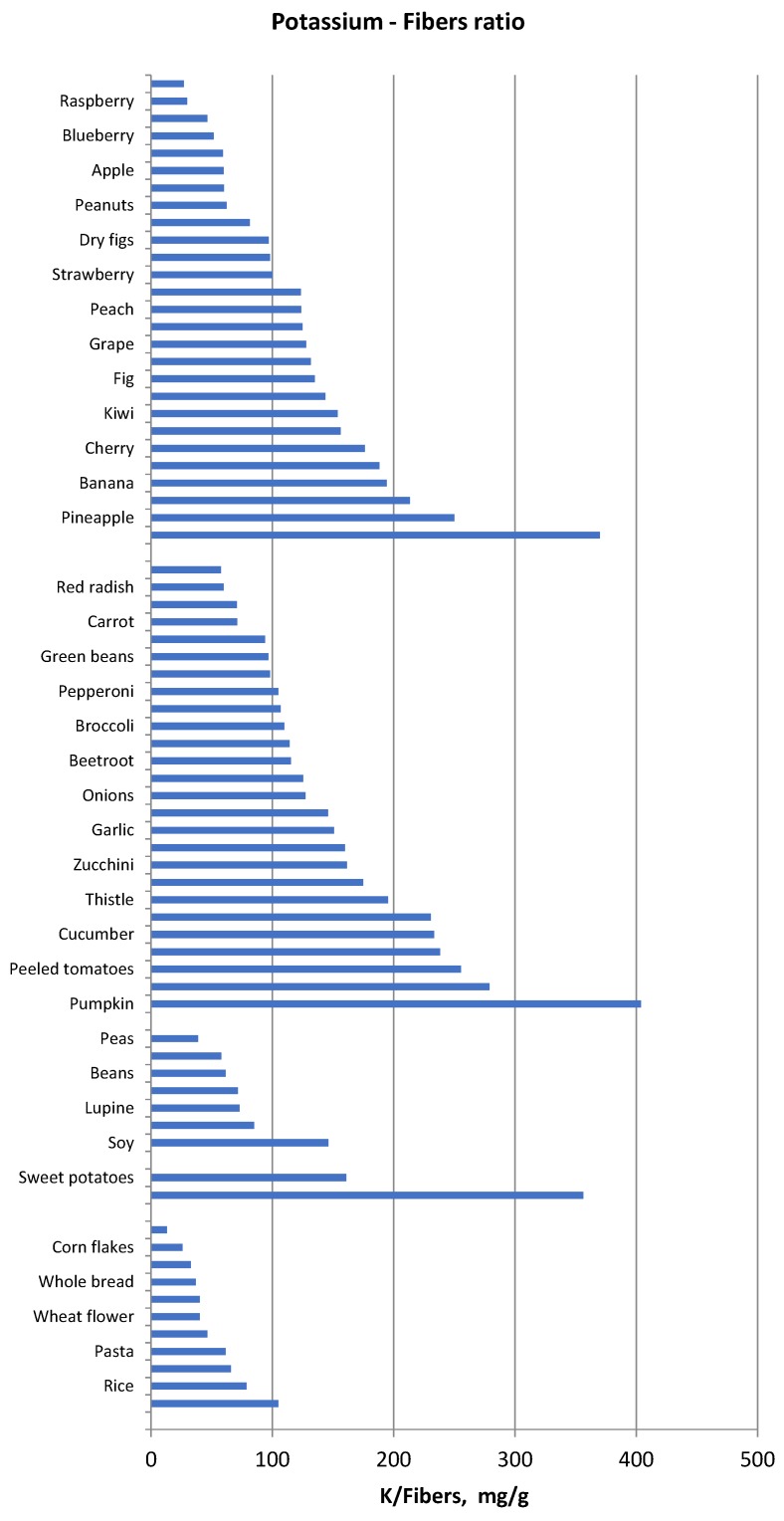
Potassium to fibre ratio (mg/g) in several food categories.

**Figure 2:**
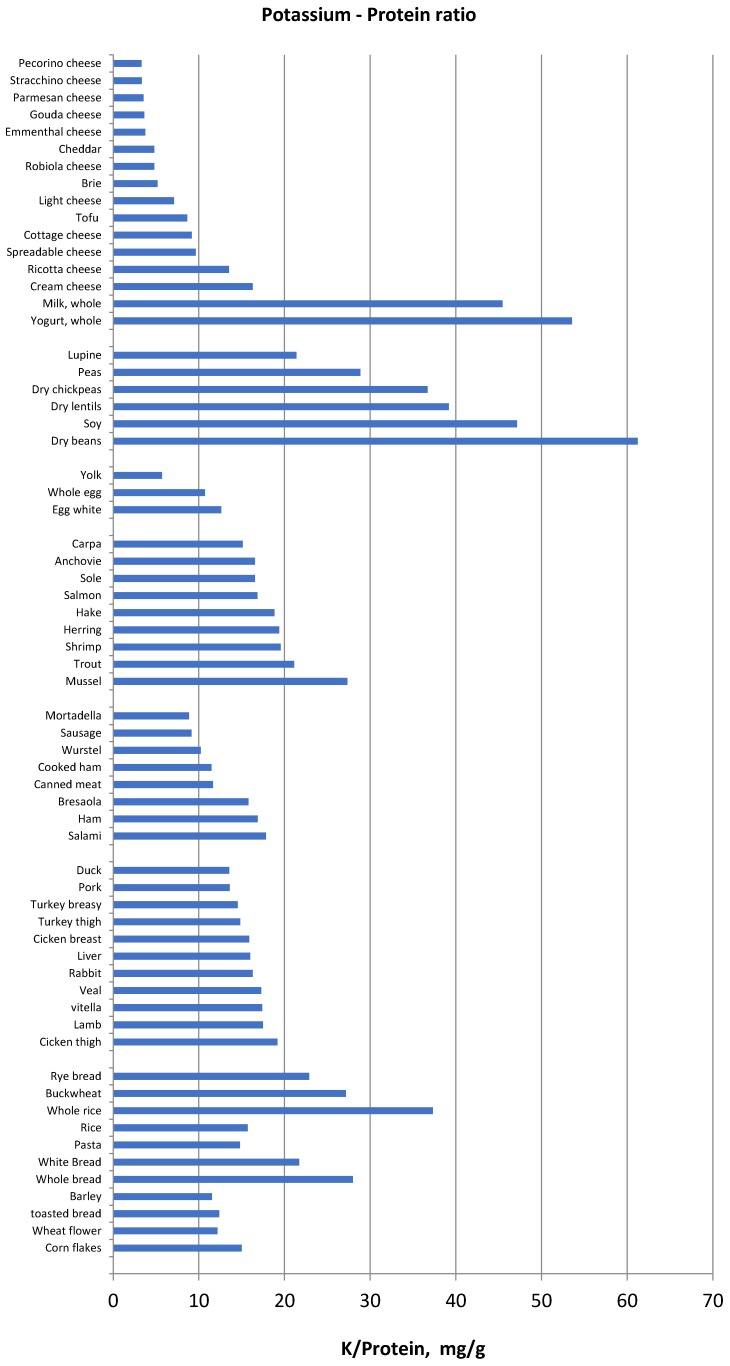
Potassium to protein ratio (mg/g) in several food categories.

Another method of food selection may be based on the potassium content normalized for unit of fibre, namely the reporting of the potassium content of vegetables and fruits also as “mg per 1 g fibre”. Foods with low potassium to fibre ratio may be allowed whereas foods with very high potassium to fibre ratio should be avoided (Figure 1). Similarly, since protein intake must be increased in haemodialysis and peritoneal dialysis patients, food selection should be addressed to reduce potassium intake without reducing dietary protein intake. Hence, reporting potassium intake per unit (g) of protein may be another method that can make it easier to limit the intake of foods that supply more potassium for the same protein intake (Figure 2) in ESRD patients.

Additional aspects useful to limit effective potassium intake is education about the use of cooking procedures (as soaking or boiling) in order to obtain food demineralization [37,38,39,40,41]: boiling is able to remove up to 60–80% of the potassium content of several raw foods (Table 4) 

Jones analyzed the mineral content of a wide variety of foods after different processing procedures. Food samples were subjected to aqueous mineral extraction after a pre-treatment which was different depending on the food group (i.e., cleaning, peeling, cutting into slices or strips, shredding etc.). They were then exposed to different water temperatures and time depending on cell type and the initial state (raw, dried etc.): for example, vegetables were placed in 2 liters of hot tap water (100–110 °F), stirred vigorously for 15–20 s and allowed to stand for a predetermined time period. Ham and hot dogs (meat group) were placed in boiling water bath, stirred and allowed to boil for 3 min. Avocado and banana from the fruit group were placed in cold tap water, stirred gently and allowed to stand for the predetermined time period. The reduction range for potassium was 59% ± 40% for vegetables, 78.5% ± 20.5% for legumes, 57% ± 41% for meats, 94% ± 3% for flours, 99% for cheddar cheese and 43% ± 16% for fruits.

Burrows and Ramer confirmed these results, finding that soaking was not effective in the leaching of significant amounts of potassium from tuberous root vegetables while the double cooking method (boil, rinse, boil again) leached more potassium than did the normal cooking method (boiling) [38]. Similar results were found also by Aiimwe et al. who showed that soaking did not change potassium content in a particular type of banana while boiling at 200 °C reduced potassium concentration from 1.4 ppm to 1 ppm after 60 min [37]. Poor results with soaking were found also by Picq et al. with various types of foods (Table 4) [40]. Preparation of food seems to be important: boiling potatoes after cubing or shredding results in a much greater loss of potassium [41].

These procedures are generally considered “negatives” as they can affect food nutritional properties, taste and appearance but giving patients appropriate instructions on how to process food after boiling (e.g., adding flavouring herbs) this obstacle can be overcome with the advantage that many restricted foods become permissible.

Finally, attention should be paid to hidden sources of potassium, such as salt substitutes and certain food additives. The former contain potassium instead of sodium and are usually recommended in hypertensive patients to reduce sodium intake and to increase potassium intake. However, in the case of treatment with RAASi and/or in patients with reduced renal function the risk of hyperkalaemia from these agents may be considerable [42]. Two categories of salt substitutes are available on the market: low-sodium salts and sodium-free salts. In the low-sodium salt the sodium chloride content must not exceed 35% (corresponding to a sodium content not exceeding 13.6 g%) and cannot be less than 20% (corresponding to a sodium content not less than 7.8%) with a potassium: sodium ratio of at least 1.5:1. In the sodium-free salts potassium content ranges between 20% and 30% and sodium content is fixed at a maximum value of 0.12%. Hence, for instance, 5–6 g of a current salt substitute can supply from 1000–1200 mg to 1500–1800 mg of extra-potassium. This represents a significant potassium load on top of the potassium derived from food intake, which is of concern in patients with reduced capacity of potassium excretion. Special attention should be paid to the use of these salt substitutes because people believe that they are safer than regular salt and they tend to use greater amounts of them. The effect of potassium-based additives on potassium burden is not widely recognized, with limited literature. Sherman and Mehta [43] found that potassium content in foods with additives varied widely and that uncooked, enhanced meat and poultry products had potassium levels up to threefold greater than similar unenhanced food products. The use of additives in packaged poultry, fish or meat foods can increase the effective dietary potassium load and of special concerns in patients with CKD, are sodium-reduced products [44,45].

For instance, additive-free products had an average potassium content <387 mg/100 g, whereas five of the 25 products with additives analyzed in that investigation contained at least 692 mg/100 g with a maximum of 930 mg/100 g [43]. Table 5 reports the most frequently used potassium-based additives and their acceptable daily intake (ADI) [43]. A temporary ADI of 3 mg/kg is currently established, while an ADI of 25 mg/kg for potassium sorbate is under evaluation [46]. Paying attention to the food labels may be useful although the quantity of potassium added as an additive is not generally available.

## 4. Low Potassium Regimens for Pragmatic Management of CKD Patients with Hyperkalaemia

Nutritional therapy in CKD is very complex, as it has to consider concomitantly the intake of protein, energy, sodium, phosphorus and potassium. Individualized nutritional education programs and regular counselling are all important aspects of clinical management, which also look to improve patients’ lifestyle. Dietary interventions require the active participation of patients and their relatives and caregivers. Recommendations or prescriptions must be simple, understandable and easy to implement in daily life by most patients.

In the Appendix, we provide a leaflet as a guide for education and counselling in the field of hyperkalaemia for patients and professionals. In our experience the use of educational tools such as brochures with food images and inserts with “traffic light” colors can be useful during counselling to summarize some essential aspects of nutritional therapy and to help patients remember instructions. Several foods within the various food groups have been color-coded. The color green identifies foods that can be safely consumed even in hyperkalaemia, orange means foods that can be consumed with caution in hyperkalaemia, while in red are foods that should be avoided in hyperkalaemic patients if possible. This results in 3 columns, which allow users to build 3 kinds of diets characterized by mild, moderate or severe potassium restriction (up to 2 g per day), recommended in the case of mild, moderate or severe chronic hyperkalaemia, respectively (Figure S1). In addition, we also provide several pragmatic suggestions meant to facilitate the application of potassium restricted diets in daily life (Figure S2). Obviously this tool, such as any other visual and practical tool, can only help in achieving excellent results if it is used within nutritional education programs appropriately applied by skilled and well trained health professionals who adapt the nutritional intervention to the individual patient. As we described above, the leaflets report a selection of foods from various food groups. It is possible that some foods are missing but boxes can be filled with other foods as needed, to adapt the tool to patients’ habits, culture, traditions and needs.

## 5. Conclusions

**Figure 3:**
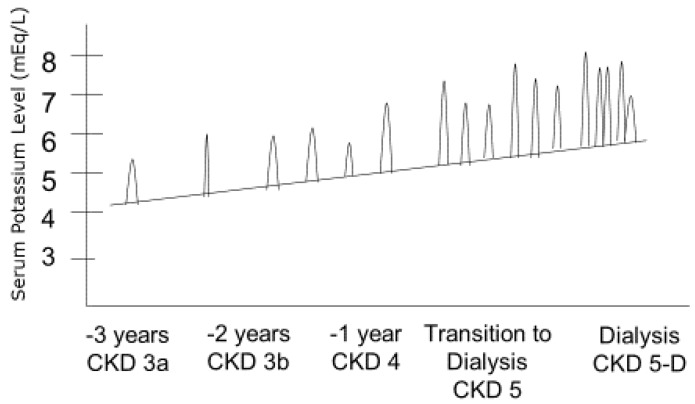
Acute on chronic and recurrent hyperkalaemia episodes by stages of chronic kidney disease (CKD).

Whereas the adequate intake of potassium is relatively high in healthy adults, i.e., 4.7 g per day, dietary potassium restriction of usually less than 3 g per day is recommended in the management of patients with CKD, especially those who tend to develop hyperkalaemia. Proper dietary counseling is imperative in CKD and ESRD patients with chronic or recurrent hyperkalaemia or those at risk of hyperkalaemia with sporadic hyperkalaemic surges as renal failure progresses (Figure 3). A more balanced and realistic approach to such overzealous restrictions would mean choosing the most beneficial sources of potassium which is only possible through a concerted education effort [46].

Special attention must be paid to avoid excess potassium intake, but together with maintenance of a high fibre intake and a low net fixed-acid load. This is an important point since constipation and metabolic acidosis are major risk factors for chronic hyperkalaemia.

To achieve a reduction of potassium load without causing a decrease in alkali or fibre intake, we recommend avoiding foods which contain excessive amount of potassium, favouring foods with low potassium content relative to fibre and protein content, providing education about the use of cooking procedures (especially boiling and soaking) in order to achieve demineralization and increasing attention to hidden sources of potassium (e.g., food additives and low-sodium salt substitutes). Using these principles, a pragmatic educational tool can be prepared to make the implementation of diets with limited potassium content and more patient-friendly in the management for CKD and ESRD patients with chronic or recurrent hyperkalaemia.

## Figures and Tables

**Table 1:** Recommended dietary potassium intake at different Stages of chronic kidney disease in adults. Adapted from Table 2 in Kalantar-Zadeh K and Fouque D [11].

	Normal kidney function (eGFR ≥ 60 *) and no proteinuria but at higher CKD risk, e.g., diabetes, hypertension, or solitary kidney	Mild to moderate CKD (eGFR 30 < 60 *) without substantial proteinuria (<0.3 g/day)	Advanced CKD (eGFR < 30 *) or any CKD with substantial proteinuria (>0.3 g/day)	Prevalent dialysis therapy, or any CKD stage with existing or imminent PEW
Dietary Potassium (g/day)	Same as recommended for the general population (4.7 g/day).	Same as the general population unless frequent or severe hyperkalaemia excursions.	<3 g/day if hyperkalaemia occurs frequently while maintaining high fibre intake.	<3 g/day target high fibre intake

* The unit for eGFR is mL/min/1.73 m^2^ body surface area (BSA). Abbreviations: CKD: chronic kidney disease, d: per day (such as in g/kg/day), eGFR: estimated glomerular filtration rate in mL/min/1.73 m^2^, PEW: protein energy wasting.

**Table 2:** Potassium content of animal-origin food, beverages, sugar and sweets and fats.

	Potassium (mg)				Potassium (mg)		
	100 g	Serving	100 kcal		100 g	Serving	100 kcal
**Meat**				**Milk and dairy**			
Chicken breast	370	370	370	Milk	150	188	234
Chicken thigh	355	355	332	Yogurt	150	188	170
Duck	290	290	182	Brie	100	50	31
Lamb	350	350	220	Cheddar	120	60	31
Liver	320	320	225	Cottage cheese	89	89	77
Pork	290	290	185	Cream cheese	150	150	84
Rabbit	360	360	261	Emmenthal cheese	107	54	27
Turkey breast	320	320	221	Gouda cheese	89	45	26
Turkey thigh	310	310	167	Parmesan cheese	120	60	30
Beef	330	330	206	Pecorino cheese	94	47	24
Veal	360	360	391	Ricotta cheese	119	119	82
**Preserved meat**				Spreadable cheese	108	54	35
Bresaola	505	253	334	Stracchino cheese	62	62	21
Canned meat	140	140	226	**Fats**			
Cooked ham	227	114	106	Butter	15	2	2
Ham	454	227	203	Cream	91	9	44
Mortadella	130	65	41	Margarine	5	1	1
Salami	473	237	123	Olive oil	0	0	0
Sausage	130	65	33	**Sugar and Sweets sand**			
Wurstel	140	70	52	Dark chocolate	300	30	55
**Fish**				Fruit ice cream	180	72	101
Anchovies	278	417	290	Honey	51	3	17
Carpa	286	429	204	Marmalade	100	5	45
Hake	320	480	451	Milk chocolate	420	42	74
Herring	320	480	148	Milk ice cream	110	44	46
Mussel	320	480	381	Sugar	2	0	1
Salmon	310	465	168	**Beverages**			
Shrimp	266	399	375	Beer	35	116	78
Sole	280	420	326	Cola	1	3	3
Trout	429	644	364	Orange juice	150	300	417
**Egg**				Red wine	110	138	145
Egg white	135	95	314	Tea	0	0	0
Whole egg	133	67	104	Wine	61	76	86
Yolk	90	31	28				

**Table 3:** Potassium content of plant-based foods.

	Potassium (mg)				Potassium (mg)		
	100 g	Serving	100 kcal		100 g	Serving	100 kcal
**Cereals and tubers**				**Pulses**			
Barley	120	60	38	Beans	650	650	625
Buckwheat	220	176	60	Dry beans	1445	723	465
Corn flakes	99	45	27	Dry chickpeas	800	400	239
Pasta	160	128	45	Dry lentils	980	490	302
Rice	110	88	30	Dry soy beans	1740	870	437
Rye bread	190	95	86	Lupine	351	351	308
Toasted bread	140	35	34	Peas	202	202	266
White Bread	176	88	64	**Fruits**			
Whole bread	210	105	86	Apple	120	180	267
Whole rice	250	200	70	Apricot	320	480	1143
Potatoes	570	1140	671	Banana	350	525	530
Sweet potatoes	370	740	425	Blackberry	260	390	722
**Vegetables**				Blueberry	160	240	640	
Asparagus	240	480	828	Cherry	229	344	603
Basil	300	600	769	Fig	270	405	574
Beetroot	300	600	1579	Grape	192	288	315
Broccoli	340	680	1259	Grapefruit	230	345	885
Carrot	220	440	667	Kiwi	400	600	909
Cauliflower	350	700	1400	Lemon	140	210	298
Celery	280	560	1400	Mango	250	375	472
Chard	286	572	1682	Melon	333	500	1009
Cucumber	140	280	1000	Orange	200	300	588
Eggplant	184	368	1227	Peach	260	390	963
Fennel	276	552	3067	Pear	130	195	325
Green beans	280	560	1556	Pineapple	250	375	625
Leeks	310	620	1069	Pomegranate	290	435	460
Lettuce	240	192	1263	Raspberry	220	330	647
Mushrooms	235	470	870	Strawberry	160	240	593
Olives	432	130	304	Tangerine	210	315	292
Onions	140	280	538	Watermelon	280	420	1867
Peeled tomatoes	230	460	1095	**Dried fruits and nuts**			
Pepperoni	210	420	955	Dried figs	1010	303	395
Pumpkin	202	404	1122	Dried plum	824	247	375
Red radish	180	360	1385	Almond	860	258	159
Rocket salad	369	295	1476	Cashew nuts	565	170	104
Spinach	530	1060	1710	Nuts	368	110	56
Zucchini	210	420	1909	Peanuts	680	204	114

**Table 4:** Potassium removal by home-based cooking methods.

Food Group/Item	Type of Treatment or Food Processing	% Potassium Content Reduction
Vegetables (15 different varieties)	Each food was placed in 2 liters of hot tap water (100–110 °F), stirred vigorously for 15–20 s and allowed to stand for a predetermined time period. Ham and hot dogs (meat group) were placed in boiling water bath, stirred and allowed to boil for 3 min. Avocado and banana from the fruit group were placed in cold tap water, stirred gently and allowed to stand for the predetermined time period [37].	59 ± 40
Fruits (8 different varieties)		43 ± 16
Legumes (5 different varieties)		78.5 ± 20.5
Meats (7 different varieties)		57 ± 41
Tuberous root vegetables *	Soaking [38]	8%
Tuberous root vegetables *	Double cooking (boil, rinse and boil again) [38]	46%
White Potato (Solanum tuberosum)	Leaching overnight after cubing [41]	0–4%
White Potato (Solanum tuberosum)	Leaching overnight after shredding [41]	2–17%
White Potato (Solanum tuberosum)	Boiling after cubing [41]	50%
White Potato (Solanum tuberosum)	Boiling after shredding [41]	69–75%
Banana (Matooke)	Soaking	No significant reduction
Banana (Matooke)	Boiling 60 min at 200 °C [39]	37%
Chocolate	Soaking [40]	16%
Potato		16%
Apple		26%
Tomato		37%
Banana	Soaking [40]	41%

* Fresh and sweet batata, cocomalanga, dasheen, eddo, black yam, white yam, yellow yam, yampi, malanga, red yautia, white yautia and yucca.

**Table 5:** Most frequently used potassium-based food additives and the corresponding acceptable daily intake (ADI).

Categories	Chemical Name	E Number (Europe)	ADI	Where to Find Them
Preservatives	Potassium sorbate	E202	3 mg/kg	Pre-cooked or long-lasting foods, powder dressings, nuts, sauces, preserved meats, stuffed pasta (tortellini, ravioli), jellies, concentrated fruit juices, processed cheeses, wine, margarine
	Potassium metabisulphite	E224	0.35 mg/kg	
	Potassium nitrate	E252	5 mg/kg	
Antioxidants and acidity regulators	Potassium citrate	E332	No limit	
	Potassium tartrate	E336	30 mg/kg	
Stabilizers, emulsifier, thickeners	Potassium alginate	E402	50 mg/kg	
	Potassium diphosphate	E450	70 mg/kg	
	Potassium triphosphate	E451	70 mg/kg	
Flavour enhancer	Potassium glutamate	E622	Not defined	
	Potassium guanylate	E628		
	Potassium inosinate	E632		

## Supplementary Materials

The following are available online at http://www.mdpi.com/2072-6643/10/3/261/s1. Figure S1: Pragmatic dietary approaches to mild, moderate and severe chronic hyperkalaemia; Figure S2: General advices for dietary management of hyperkalaemia.

## Abbreviations

CKD	Chronic Kidney Disease
RAAS	Renin-Angiotensin-Aldosterone System
ESRD	End Stage Renal Disease
RDA	Recommended Daily Allowance
NDD	Non-Dialysis Dependent
NKF	National Kidney Foundation
MBD	Mineral Bone Disease
HF	Heart Failure
LPD	Low Protein Diet
ADI	Acceptable Daily Intake
BSA	Body Surface Area
DPI	Dietary Protein Intake
EAA	Essential Amino-Acids
eGFR	estimated Glomerular Filtration Rate
HBV	High Biologic Value (referred to protein)
HTN	Hypertension
IBW	Ideal Body Weight
KA	Ketoacids (keto-analogues of amino-acids)
P	Phosphorus
PD	Peritoneal Dialysis
RKF	Residual Kidney Function
sHPT	secondary Hyperparathyroidism
